# The paramyxovirus polymerase complex as a target for next-generation anti-paramyxovirus therapeutics

**DOI:** 10.3389/fmicb.2015.00459

**Published:** 2015-05-12

**Authors:** Robert Cox, Richard K. Plemper

**Affiliations:** Institute for Biomedical Sciences, Petit Science Center, Georgia State University, Atlanta, GAUSA

**Keywords:** Paramyxovirus, RNA-dependent RNA polymerase, antiviral therapy, nucleoside analogs, allosteric inhibitor

## Abstract

The paramyxovirus family includes major human and animal pathogens, including measles virus, mumps virus, and human respiratory syncytial virus (RSV), as well as the emerging zoonotic Hendra and Nipah viruses. In the U.S., RSV is the leading cause of infant hospitalizations due to viral infectious disease. Despite their clinical significance, effective drugs for the improved management of paramyxovirus disease are lacking. The development of novel anti-paramyxovirus therapeutics is therefore urgently needed. Paramyxoviruses contain RNA genomes of negative polarity, necessitating a virus-encoded RNA-dependent RNA polymerase (RdRp) complex for replication and transcription. Since an equivalent enzymatic activity is absent in host cells, the RdRp complex represents an attractive druggable target, although structure-guided drug development campaigns are hampered by the lack of high-resolution RdRp crystal structures. Here, we review the current structural and functional insight into the paramyxovirus polymerase complex in conjunction with an evaluation of the mechanism of activity and developmental status of available experimental RdRp inhibitors. Our assessment spotlights the importance of the RdRp complex as a premier target for therapeutic intervention and examines how high-resolution insight into the organization of the complex will pave the path toward the structure-guided design and optimization of much-needed next-generation paramyxovirus RdRp blockers.

## Introduction

Paramyxoviruses are enveloped, non-segmented and single-stranded RNA viruses with negative genome polarity (NNRV) in the order *Mononegavirales*, which also includes the *Bornaviridae*, *Filoviridae*, and *Rhabdoviridae* families. The paramyxoviruses encompass major human and animal pathogens such as respiratory syncytial virus (RSV), measles virus (MeV), mumps virus (MuV), and Newcastle disease virus (NDV). The family is organized into two subfamilies, the Pneumovirinae and the Paramyxovirinae. While RSV belongs to the former subfamily, MeV, MuV, NDV, and the newly emerged Hendra and Nipah viruses are all part of the Paramyxovirinae.

All paramyxoviruses spread through the respiratory route and predominantly cause acute disease, and several members of the family are extremely contagious. For example, MeV is considered the most infectious viral pathogen identified to date (Kelly et al., 2002; Centers for Disease and Prevention, 2012a). Although vaccines are available for some paramyxoviruses, much-needed effective antiviral therapeutics for post-exposure prophylaxis and improved disease management are lacking. Moreover, vaccine prophylaxis against several clinically highly significant members of the family is still unavailable despite major past research efforts.

Respiratory syncytial virus, for instance, is the leading cause of infant mortality from viral respiratory disease and responsible for over 120,000 infant hospitalizations per year in the U.S. alone. Whereas clinical symptoms of paramyxovirus disease are frequently based on immunopathogenicity rather than directly virus induced (Hall et al., 1971; Auwaerter et al., 1999), in the case of RSV infection higher viral loads serve as a predictor of RSV lower respiratory tract infection in infected infants (DeVincenzo et al., 2005). Among hospitalized RSV-infected children less than 2 years of age, viral load on day three of hospitalization was also associated with a requirement for intensive care and respiratory failure (El Saleeby et al., 2011). These findings spotlight a window of opportunity for improved RSV disease management through therapeutics, but post-exposure prophylaxis may be the only viable indication against other clinically significant members of the family. For example, we propose that a combined prophylactic and post-exposure therapeutic anti-measles platform may be required to ultimately prevail in a prolonged endgame of gaining global measles control (Plemper and Snyder, 2009; Plemper and Hammond, 2014). Despite major educational efforts, herd immunity remains too low to interrupt endemic MeV transmission in large areas of Western Europe due to parental concerns against vaccination (Larson et al., 2011; Saint-Victor and Omer, 2013), and local pockets with low vaccination coverage increasingly sustain transmission of imported virus in the U.S. (Centers for Disease and Prevention, 2012b).

Executing essential and virus-specific enzymatic activities, the viral RdRp complex represents an attractive, albeit underexplored, target for therapeutics. This review will summarize current insight into the spatial organization and function of the paramyxovirus RdRp complex and assess candidate druggable targets within the complex based on the available structural information and experimental therapeutics.

## Components of the RdRp Complex

The overall genome organization and fundamental principles for genome replication and transcription are conserved between different paramyxoviruses and, to some extend, all NNRV. Throughout the virus replication cycle, the genome exists as a unique ribonucleoprotein complex, the nucleocapsid (NC), which is composed of the genomic RNA completely sequestered by copies of the viral NC (N) protein. Only the NC can serve as a template for RNA synthesis by the RdRp complex, which consists of the viral large (L) and phospho-(P) proteins in addition to host co-factors. The L protein contains all enzymatic activities exercised by the complex, while P acts as an essential cofactor. The NC, P, and L core complex functions as both replicase and transcriptase. Although present in all paramyxoviruses, in most cases only homotypic N, P, and L combinations, in which each component is derived from the same paramyxovirus family member, are bioactive (Smallwood and Moyer, 2004; Dochow et al., 2012). Functional studies on N, P, and L have furthermore confirmed that each of the RdRp components can individually and differentially affect the processes of mRNA synthesis and genome replication (Perlman and Huang, 1973; Chen et al., 1997; Fearns et al., 1997; Hwang et al., 1999; Galloway and Wertz, 2008, 2009; Harouaka and Wertz, 2009).

## Transcriptase Activity

**FIGURE 1:**
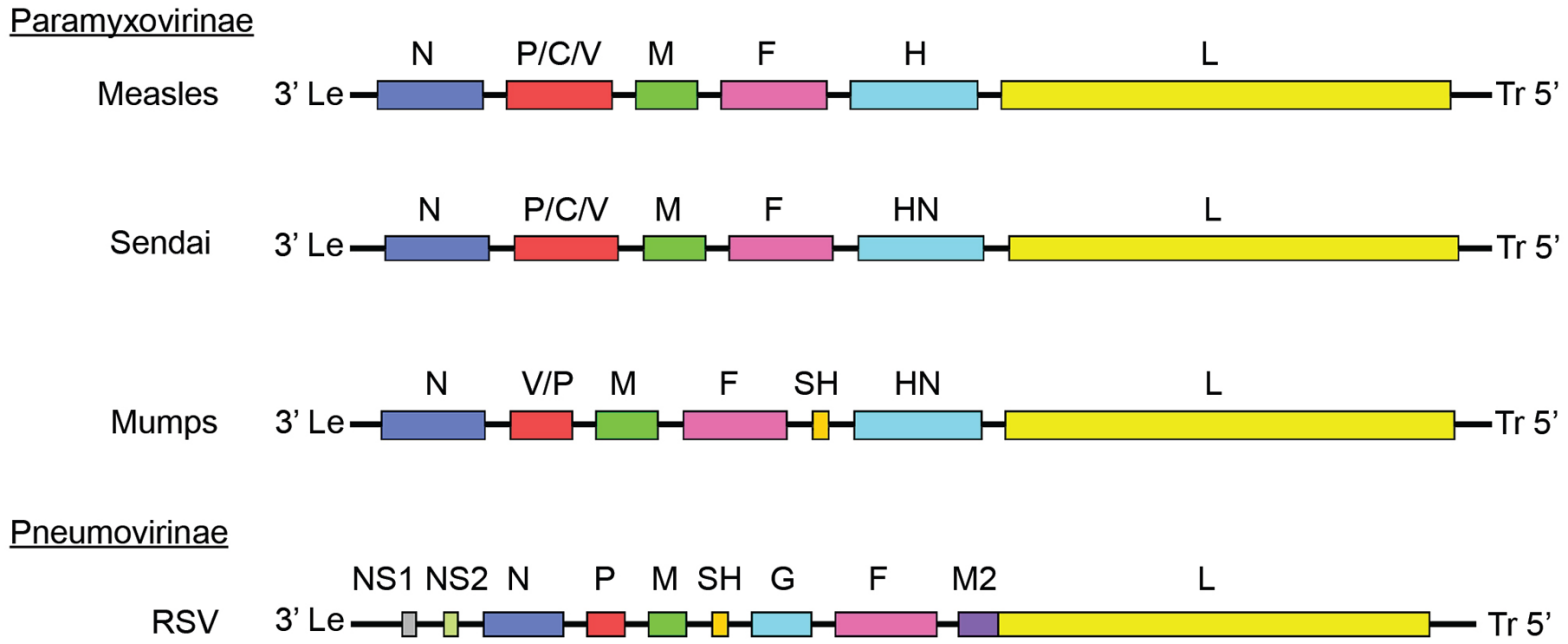
**Paramyxoviridae genome organization**. Genomes of different members of the Paramyxoviridae family. Members of the Paramyxovirinae subfamily include measles virus (MeV), mumps virus (MuV), and Sendai virus. Respiratory syncytial virus (RSV) is a member of the pneumovirinae subfamily. Genome organization and numbers of open reading frames differ between members of the family, but all paramyxoviruses use a core RdRp composed of the NC, P, and L.

**FIGURE 2:**
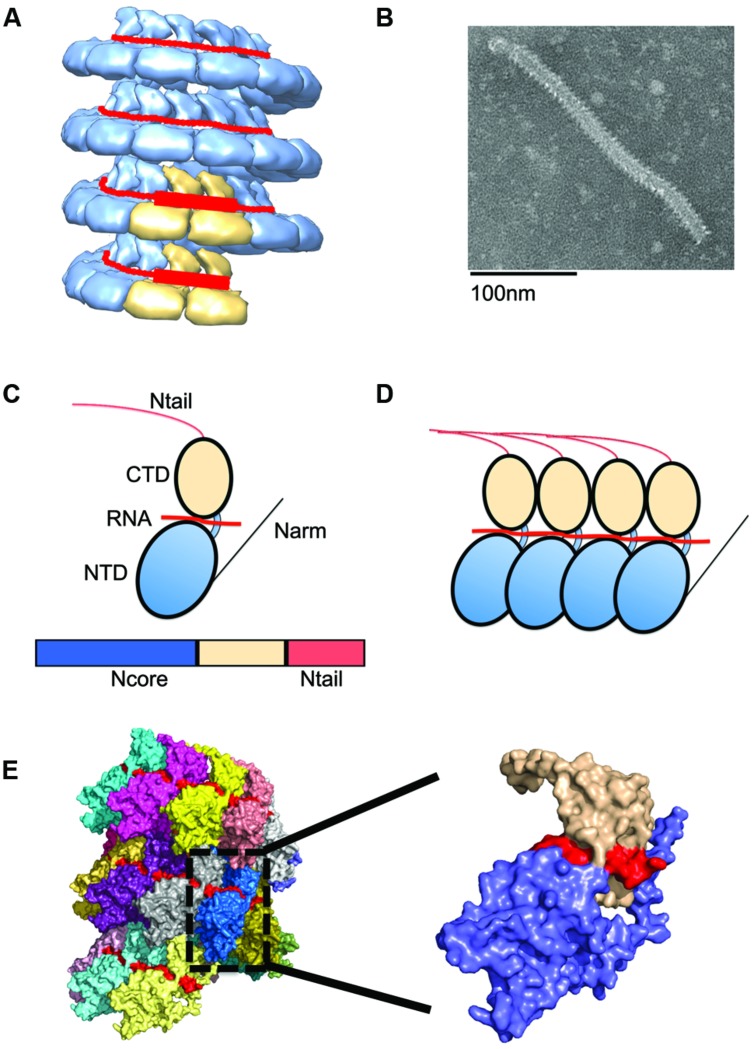
**Nucleocapsid (NC) architecture. (A)** Illustration of the bipartite promoter organization by example of the MuV NC (EMD-2630; Cox et al., 2014). N protein protomers encapsidating promoter regions in genomic RNA are highlighted in tan. **(B)** Typical ‘herring-bone’ structure of the paramyxovirus NC. **(C)** The paramyxovirus N protein is composed of two core domains (NCore), NTD (blue), and CTD (tan). A schematic of the N protein is shown below the cartoon model. For paramyxoviruses, the extreme C-terminus of N, N_Tail_, extends out from the NC and interacts with P. **(D)** Extensions from these domains, N-arm and C-arm interact with neighboring N subunits in the helical NC. **(D,E)** N subunits assemble side by side along the genomic RNA. **(E)** The structure of the RSV NC showing the encapsidated RNA in red. PDB code 4BKK (Bakker et al., 2013). The insert shows an enlargement of a single N protein protomer, color coded as in **(C)**.

Upon entry into the host cell, virion uncoating separates genome and viral envelope and releases the NC along with the attached RdRp into the cytoplasm. Once in the cytoplasm, encapsidated genomic RNA serves as the template for both transcription and replication. Leader (Le) and trailer (Tr) sequences are located at the 3′- and 5′-termini of the genome, respectively, and harbor the genomic and antigenomic promoter elements, (**Figure 1**). RSV contains a linear genomic promoter that spans the first 12 residues of the genome (Noton et al., 2014), while members of the paramyxovirinae subfamily contain bipartite promoters (**Figure 2A**; Pelet et al., 1996; Murphy et al., 1998; Tapparel et al., 1998). Encapsidation is essential for the assembly of a functional bipartite promoter, since distinct promoter elements are juxtaposed only in the helical NC. Consensus sequences that are involved in transcription initiation, polyadenylation, and transcription termination of individual genes are located at the beginning and end of each gene. In transcriptase mode, RdRp initiates synthesis of the first functional mRNA at the first gene-start consensus sequence. The nascent mRNA is capped and methylated by L, and then the full mRNA transcript is generated (Moyer and Banerjee, 1975; Ogino et al., 2005; Ogino and Banerjee, 2007). At the end of each open reading frame, RdRp recognizes a signal for non-templated polyadenylation, followed by release of the viral mRNA (Lamb and Parks, 2007). Next, the complex proceeds over the intergenic sequence and reinitiates transcription at the next downstream transcription start site. However, reinitation is only partially efficient, which results in a transcription gradient – the synthesis of progressively less of each viral mRNA as the RdRp advances along the template – that is characteristic for all members of the mononegavirales.

## Replicase Activity

Although transcription and replication use the same viral proteins, they are two distinct processes. While transcription by some NNRV RdRps can occur *in vitro* in the presence of NC, the correct salts, and ribonucleotides (Emerson and Wagner, 1972; Davis and Wertz, 1982), genome replication requires ongoing N protein synthesis, since the nascent genomic or antigenomic RNA is encapsidated concomitantly. In order to switch from transcription to replication, a sufficiently large amount of N protein must be available in order to encapsidate the newly synthesized genomes and antigenomes. In fact, in the case of the paramyxovirinae at least, the intracellular N protein pool serves as a major driver inducing the switch from initial transcription to replication (Baker and Moyer, 1988; Horikami et al., 1992). For the pneumovirinae, however, the available N protein pool alone is not responsible for the shift to replicase functionality, since increased levels of RSV N enhanced antigenome synthesis, but had no effect on transcription levels in RSV minireplicon experiments (Fearns et al., 1997). When in replicase mode, RdRps derived from either subfamily ignore all *cis*-acting signals, such as polyadenylation sites, to produce full-length genomic RNA copies.

The array of distinct enzymatic activities of the RdRp complex and the highly dynamic protein–protein and protein–RNA interactions that are required for bioactivity provide rich opportunity for therapeutic interference. As a basis for discussing individual druggable targets, we will illuminate the role of the viral protein components in RdRp complex assembly and function.

## Nucleocapsid Protein

The paramyxovirus NC shows a characteristic herringbone structure in electron micrographs (**Figure 2**). Despite this defined appearance, the NC remains flexible with variations in pitch and helical symmetry parameter along its length, which may be required to allow the polymerase complex to access the encapsidated RNA without disassembling the helix (Heggeness et al., 1980; Egelman et al., 1989; Bhella et al., 2002, 2004).

**FIGURE 3:**
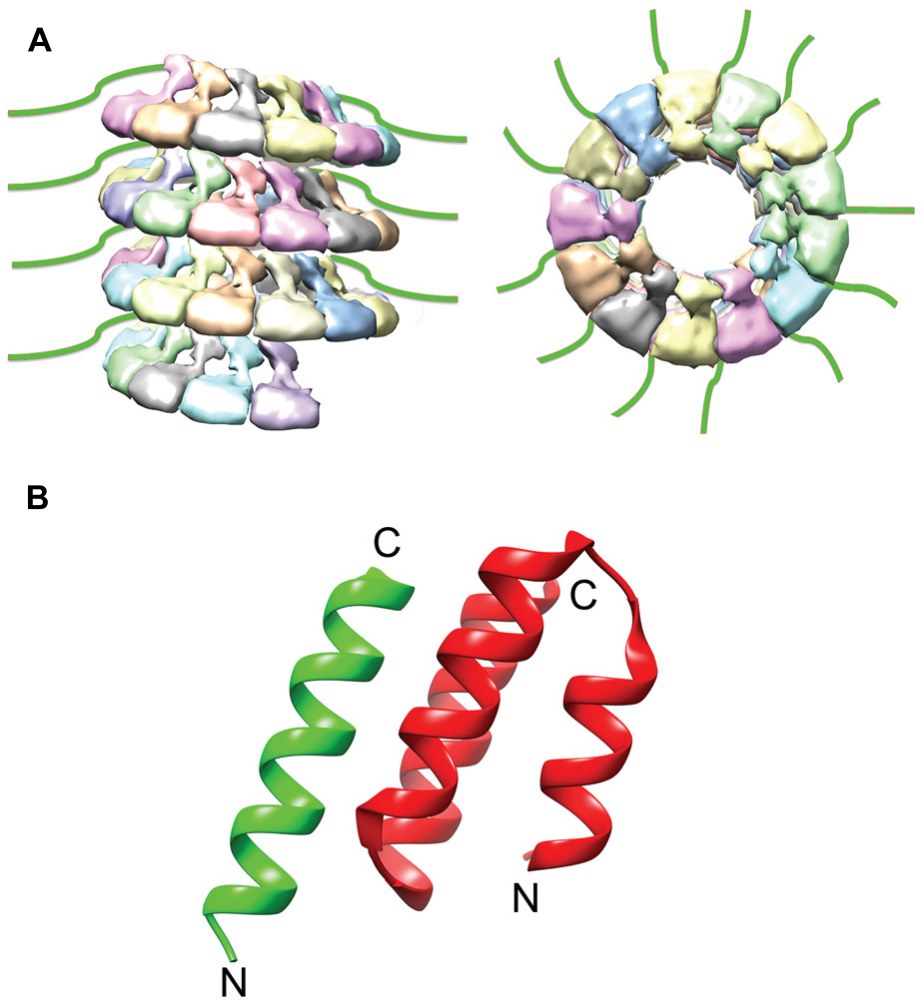
**N_Tail_ and N_Tail_–P_CTD_ Interaction. (A)** The extreme C-terminal region of N, N_Tail_ (green), is highly flexible and extends outward from the assembled NC (Jensen et al., 2011). **(B)** A short helix in N_Tail_ (green) is involved in binding the C-terminal NC binding domain of P (red). The N- and C- termini are labeled (PDB code 1T6O; Kingston et al., 2004a). The NC model was modified from the mumps NC structure (EMDataBank access code EMD-2630; Cox et al., 2014).

N subunits in the NC are assembled side-by-side and parallel along the length of the RNA to form a highly unique protein–RNA complex, in which the viral RNA is entirely sequestered by the N protein (**Figure 2**; Tawar et al., 2009). Each N protomer is organized into an N-terminal (NTD) and C-terminal (CTD) core domain, which are connected through a hinge region (**Figure 2**). Both the NTD and CTD interact laterally with adjacent subunits. The RNA interaction site is positioned at the NTD/CTD interface, forming a basic surface groove into which the RNA threads belt-like along the outside of the NC (**Figure 2**). A crystal structure of the RSV N domains was recently solved and reveals parallel layers of RSV N:RNA rings (El Omari et al., 2011). The NTD and CTD of each N subunit have N- and C-terminal extensions, termed N-arm (residues 1–28) and C-arm (residues 360–375), respectively, which attach to neighboring N subunits (Tawar et al., 2009). Of these, the N-arm is considered to provide the main stabilizing lateral N–N interaction. However, weaker top–bottom interactions may likely exist between different rungs of the helical NC. Between layers, the RSV N subunits engage in weak contacts between the N-terminal domains of one layer and the C-terminal domains of the adjacent lower layer (El Omari et al., 2011). In the RSV N:RNA structure, the C-arm lies above the CTD, occupying space between consecutive turns of the helical NC. However, for other paramyxoviruses, the extreme C-terminus of N, called N-tail, is displayed on the exterior of the NC (**Figure 3**; Jensen et al., 2011; Communie et al., 2013b). Removal of the N-tail causes the NC to rigidify, rendering it more compact and biologically inactive (Schoehn et al., 2004).

## Phosphoprotein

**FIGURE 4:**
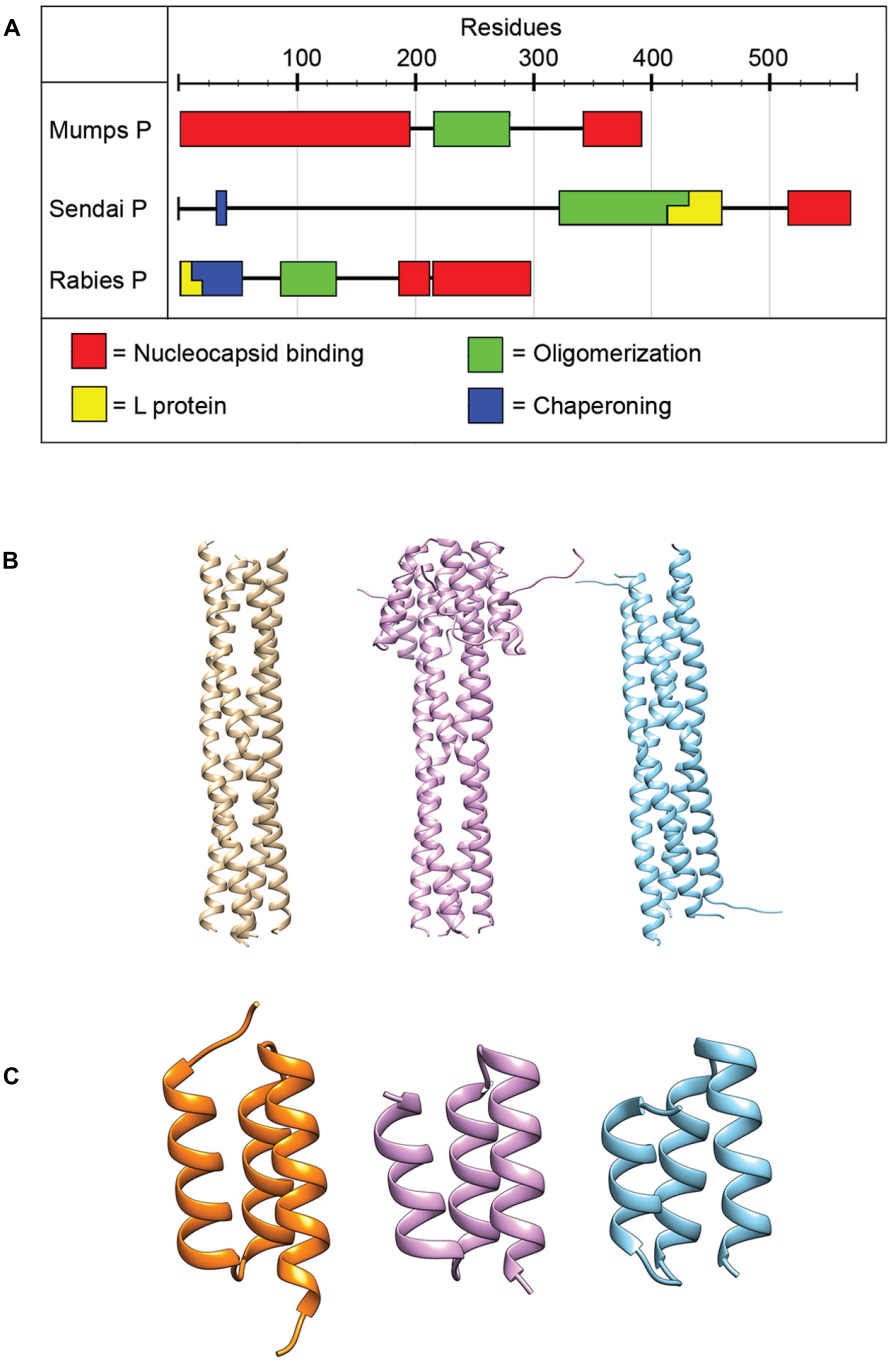
**Phosphoprotein organization and structure. (A)** The P proteins of NNRVs vary in length and domain organization. All NNRV P form oligomers. **(B)** The oligomerization state of all paramyxovirus P proteins is a tetramer. Tetrameric structures for NiV, MeV, and MuV are shown from left to right (PDB codes 4GJW, 4BHV, and 4EIJ for NiV, MeV, and MuV, respectively; Communie et al., 2013a; Cox et al., 2013; Bruhn et al., 2014). **(C)** The structures of the C-terminal NC binding domain of several paramyxovirus P have been solved and are highly conserved structurally. Structures shown from left to right are for HeV, MeV, and MuV. (PDB codes 4HEO, 3BBZ, and 1OKS, respectively; Johansson et al., 2003; Kingston et al., 2008; Communie et al., 2013b).

The P protein lacks inherent catalytic activity, but is an essential co-factor of the RdRp complex. Although required for the replication of all NNRVs, paramyxovirus P proteins vary greatly in length and sequence (**Figure 4**; Tarbouriech et al., 2000b; Karlin et al., 2003; Ding et al., 2004, 2006; Mavrakis et al., 2004; Ivanov et al., 2010). P performs a dynamic range of different functions in the virus replication cycle. The protein is thought to properly position the L protein for RNA synthesis (Kingston et al., 2004a,b, 2008), interact with the NC template (Habchi et al., 2011; Longhi, 2012; Communie et al., 2013b; Cox et al., 2014), and chaperone newly synthesized, RNA-free N protein (N^0^) to the nascent viral RNA during replication (Mavrakis et al., 2006; Chen et al., 2007; Yabukarski et al., 2014). Reflecting these diverse tasks, P shows a modular organization of different functional domains separated by flexible linker regions (Tarbouriech et al., 2000a; Karlin et al., 2003; Blanchard et al., 2004; Llorente et al., 2006). Structures for individual domains of several NNRV P proteins have been solved previously (**Figure 4**; Tarbouriech et al., 2000b; Ding et al., 2004, 2006; Mavrakis et al., 2004; Ivanov et al., 2010), but the structure of a full-length paramyxovirus P has yet to be determined.

All NNRV P proteins contain a motif in their central region known as the oligomerization domain (Tarbouriech et al., 2000b; Ding et al., 2006; Gerard et al., 2009; Ivanov et al., 2010; Communie et al., 2013a; Cox et al., 2013; Bruhn et al., 2014). In addition, all NNRV P form homo-oligomers, but their lengths and oligomerization states vary (Tarbouriech et al., 2000b; Gerard et al., 2009; Communie et al., 2013a; Cox et al., 2013; Bruhn et al., 2014). However, the tetramer is considered to represent the physiological oligomer of paramyxovirus P proteins (Tarbouriech et al., 2000a,b; Cox et al., 2013; Bruhn et al., 2014). Proper P oligomerization is required for its role in both transcription and replication (Tarbouriech et al., 2000b; Kolakofsky et al., 2004; Chen et al., 2006), and structures of the oligomerization domains for several paramyxoviruses have been solved (**Figure 4**; Tarbouriech et al., 2000b; Communie et al., 2013a; Cox et al., 2013; Bruhn et al., 2014).

Since the L protein alone is unable to bind efficiently to the NC, a key function of P is to position the RdRp on the NC and ensure continued contact between the RdRp and the template as the complex progresses along the NC. According to the precedent set by vesicular stomatitis virus (VSV), an NNRV of the rhabdovirus family, after P binding to its NC, N-terminal L binding domains protrude outward and may serve as a latch to position L (Emerson and Schubert, 1987; Morin et al., 2012). Based off of previously solved crystal structures, it is possible that paramyxovirus P proteins bind L in a similar fashion (Tarbouriech et al., 2000a,b; Cox et al., 2013; Bruhn et al., 2014), since in all of these structures N-terminal domains are proposed to protrude outward.

**FIGURE 5:**
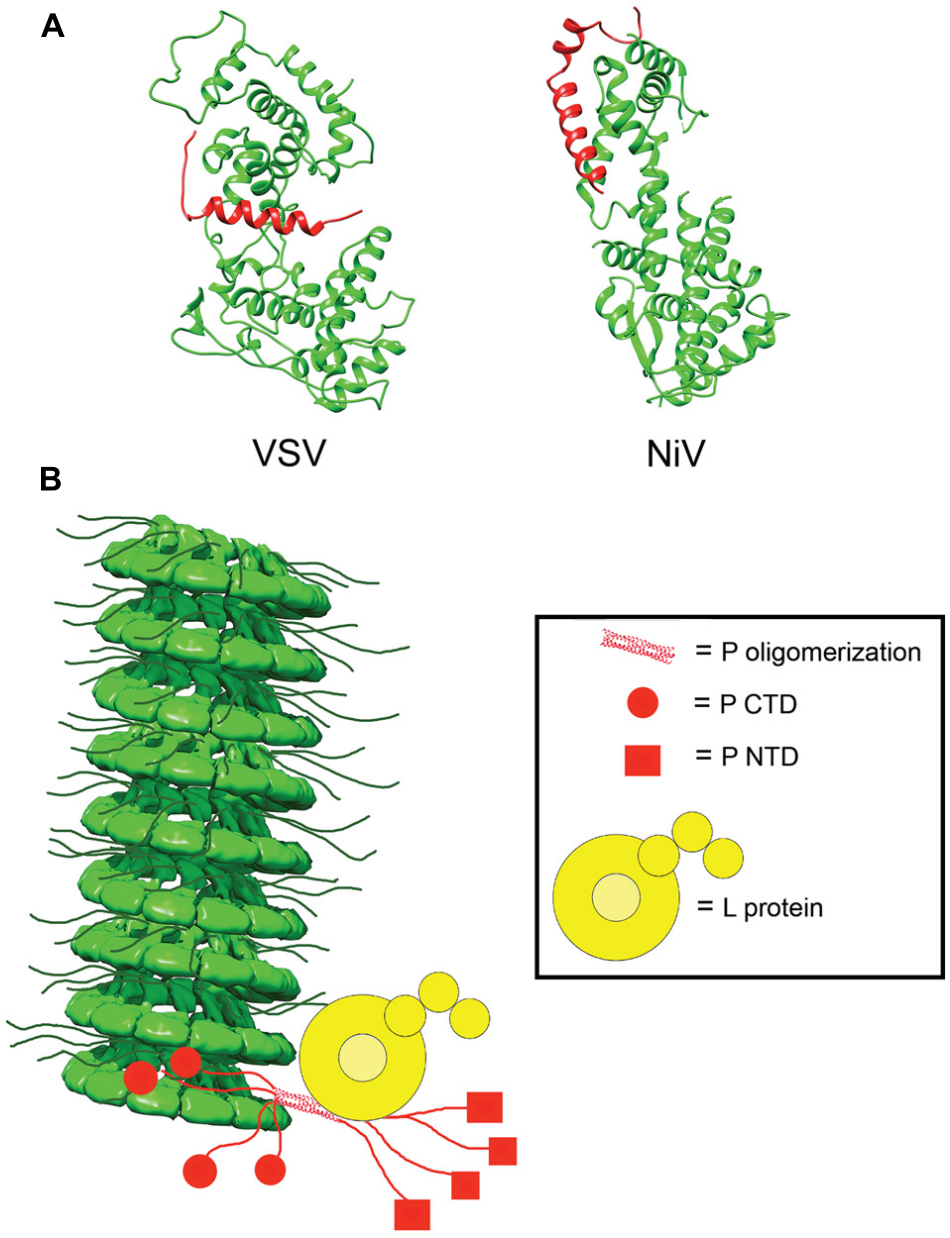
**N^0^P and model of the RdRp complex. (A)** Structures of the N^0^P complex of vesicular stomatitis virus (VSV) and NiV have been solved. P (red) acts to prevent premature encapsidation and oligomerization of N (green; PDB codes 3PMK and 4CO6 for VSV and NiV, respectively; Leyrat et al., 2011; Yabukarski et al., 2014). **(B)** Model of the interactions between N, P, and L in the viral RdRp complex. P anchors L to the NC via interactions in P_CTD_ (red circle) and correctly positions the polymerase to begin synthesizing the viral RNA. The NC model was modified from the mumps NC structure (EMD-2630; Cox et al., 2014).

In the absence of other viral proteins, N has a strong tendency to polymerize and to encapsidated non-viral cellular RNAs. To prevent non-productive N polymerization, P acts as a molecular chaperone and complexes RNA-free N^0^ forms in N^0^–P structures (Mavrakis et al., 2006; Chen et al., 2007; Leyrat et al., 2011; Yabukarski et al., 2014). In addition to blocking premature oligomerization, the N^0^–P complexes inhibit non-specific encapsidation of cellular RNA and keep N^0^ soluble (Masters and Banerjee, 1988; Chen et al., 2007; Leyrat et al., 2011; Yabukarski et al., 2014). Crystal structures for VSV and NiV N^0^–P complexes have been solved (**Figure 5**; Leyrat et al., 2011; Yabukarski et al., 2014). A comparison between the structures of the VSV and NiV N^0^–P complexes reveals a common mechanism of N^0^ chaperoning (Leyrat et al., 2011; Yabukarski et al., 2014). In both cases, the N-terminal N^0^-binding region of P prevents N polymerization by occupying the binding cavity for the N-arm and C-arm of adjacent N subunits. Proper encapsidation of the newly synthesized RNA genome requires the delivery of soluble RNA-free N^0^ to the site of RNA synthesis (Yabukarski et al., 2014). The N^0^–P complex can bind to the NC, but little is known about the reaction by which N^0^ is transferred from P to the RNA. Conceivably, an N^0^–P complex may bind to the NC template through the C-terminal NC binding domain of P, and the intrinsic flexibility of P may properly position and orient the N^0^ molecule within the replication complex and deliver it to the nascent RNA.

The interaction of P with the NC is mediated through a well-conserved nucleocapsid binding domain (NBD), which is located toward the C-terminal end of the P protein (**Figure 4**; Johansson et al., 2003; Kingston et al., 2004b). Structures of the NBDs of several paramyxoviruses complexed with their interacting domain in N have been solved (**Figure 3**; Gely et al., 2010; Habchi et al., 2011; Communie et al., 2013b). In the case of MeV, the P binding site is located near the C-terminus of the N protein, close to the end of the 125-residue N-tail domain (Kingston et al., 2004a). How the RdRp accesses the encapsidated RNA is unclear. One possibility is that a hinge movement of the NTD with respect to the CTD results in a transient opening of the groove and exposure of encapsidated nucleotides during RNA readout. In this model, the N protein acts as a helicase, dissociating the transient double-stranded RNA segment during procession of RNA synthesis along the genome (Tawar et al., 2009). The L protein, the P protein, or the L–P complex might be able to induce this conformational change (Cox et al., 2014). Physical movement of the polymerase along the NC during RNA synthesis has furthermore been hypothesized to involve the continuous attachment and release of the P NBD domains from its counterpart in the N tails (Kingston et al., 2004b), resulting in “cartwheeling” of the P–L complexes along the NC (**Figure 5**; Kingston et al., 2004b). In this model, the N-tail sections exposed on the outside of the NC are thought to serve as essential anchor points for recruitment of the polymerase complex (Curran et al., 1993; Kingston et al., 2004a,b).

Supporting this hypothesis, minireplicon reporter studies of truncated SeV and MeV N lacking the P binding domains in N suggested that N-tail truncated NCs cannot serve as a template for the RdRp, thus spotlighting a possible essential function for the N tail-P interaction in polymerase loading and/or advancement (Curran et al., 1993; Zhang et al., 2002). Strikingly, however, further truncation of the N-tail beyond the P interaction region largely restored template function of the NC in an MeV minigenome system (Krumm et al., 2013). This observation demonstrated that the P interaction with the N-tail is dispensable for initial productive loading of the RdRp onto the NC or subsequent advancement of the complex along the template (Krumm et al., 2013). Supported by RdRp activity experiments obtained with negative and positive sense replicon constructs, N-tail-independent RdRp loading appears not to be restricted to transcriptase configuration, but is also applicable to the replicase complex (Krumm et al., 2013). Interestingly, a recent characterization of the related MuV P protein revealed that its interaction with NC likewise does not depend on the N-tail but can be mediated by direct contacts between MuV P and the NTD core (Kingston et al., 2004a; Cox et al., 2013, 2014). Interestingly, this MuV P-NTD interaction would bring the associated L protein into close proximity of the encapsidated RNA. Taken together, these recent discoveries indicate that the initial tethering of the RdRp complex to the NC template is independent of the P and N tail interaction. Rather, cycles of N-tail to P binding and release may be necessary to stabilize the RdRp–NC complex as the polymerase progresses along the genome (Curran and Kolakofsky, 1999; Kolakofsky et al., 2004; Krumm et al., 2013).

## Large Protein

**FIGURE 6:**
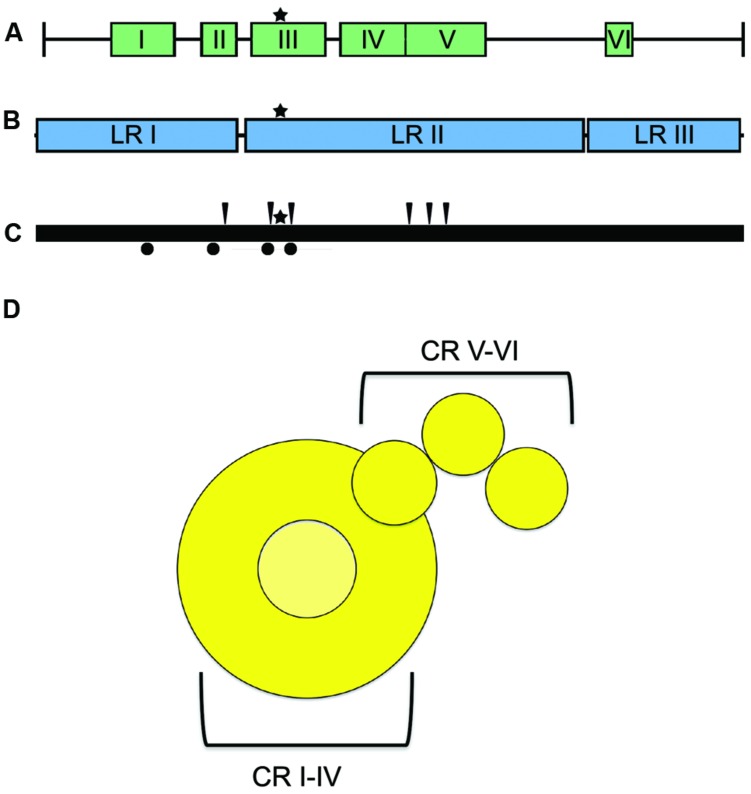
**L protein domain organization and architecture. (A)** The L protein is composed of six conserved regions (CR), I–VI, each containing separate functions (Sidhu et al., 1993). **(B)** Insertion analysis has shown that L can be further grouped into three large regions (LRI-III; Dochow et al., 2012). Star symbols mark the proposed position of key residues near the catalytic site for phosphodiester bond formation in CR III. **(C)** Positions of L mutations allowing for resistance to ERDRP-0519 in MeV (black arrows) and CDV (black circles) (Krumm et al., 2014). Mutations map to areas proximal to the active site for phosphodiester bond formation (black star) **(D)** Cartoon resembling the low-resolution organization of the VSV L protein revealed by electron microscopy analysis (Rahmeh et al., 2010). The cartoon representation is labeled with the proposed positions of the CR domains.

The large (L) protein harbors the catalytic centers required for RNA synthesis, mRNA capping, and mRNA polyadenylation (Emerson and Yu, 1975; Hamaguchi et al., 1983; Gupta et al., 2002; Ogino et al., 2005). Bioinformatics analyses have identified six conserved domains (CR I to CR VI) in NNRV L proteins that are connected by variable linker regions (**Figure 6**; Poch et al., 1988, 1990; Svenda et al., 1997). However, the precise roles for each of these L domains in RdRp function are still largely unclear. CR I has been implicated in L oligomerization (Cevik et al., 2003, 2004; Smallwood and Moyer, 2004) and L–P interactions (Horikami et al., 1994; Holmes and Moyer, 2002; Cevik et al., 2003, 2004; Chattopadhyay and Shaila, 2004), CR III is involved in phosphodiester bond formation for RNA polymerization (Malur et al., 2002b), and CR VI contains methyltransferase activity (Poch et al., 1990; Ferron et al., 2002). A conserved GXXT_n_HR motif in CR V of VSV L is thought to mediate unusual capping of the viral mRNAs through transfer of 5′-monophosphate-mRNA onto GDP (Ogino and Banerjee, 2007; Li et al., 2008). However, paramyxovirus L proteins may possess traditional guanylyltransferase activity, since Rinderpest virus RdRp complexes reportedly form covalent guanosine monophosphate-L intermediates *in vitro* (Gopinath and Shaila, 2009). In addition, a conserved guanylyltransferase consensus motif required for transcriptase activity was identified in the C-terminal region of the L protein of human parainfluenza virus (HPIV) type 2 (Nishio et al., 2011).

Consistent with L having a modular arrangement of functional domains, also studies of purified L proteins of NNRV by electron microscopy supported a linear organization of structural domains (**Figure 6**; Rahmeh et al., 2010). Analysis of the L protein of MeV has furthermore revealed that the protein can be split into distinct fragments that are capable of reconstituting RdRp bioactivity through *trans*-complementation (Duprex et al., 2002; Dochow et al., 2012). This study showed that MeV L is composed of at least two independently folding-competent domains. Consistent with these findings, sequence alignments of different morbillivirus L proteins had previously suggested two linker domains that separate three large regions (LR I to LR III; **Figure 6**; Mcllhatton et al., 1997; Duprex et al., 2002). Of these, LR I harbors CR I and II, LR II contains CR III-CR V, and LR III is considered to encompass the methyltransferase and, possibly, the recently proposed guanylyltransferase functions of CR VI. L proteins of MeV and rinderpest virus tolerated polypeptide insertions into the LR II/LR III but not the LR I/LR II junction (Duprex et al., 2002; Brown et al., 2005), consistent with at least a two-domain organization. Additional domain intersections may well exist in the paramyxovirus L protein.

In addition to the mandatory interaction with P, Sendai virus L was shown to exist as an oligomer in the RdRp complex (Smallwood et al., 2002). Homo-oligomerization was furthermore proposed for MeV and human parainfluenzavirus type 3 L proteins, and in all cases the L–L interaction domain was proposed to reside in the N-terminal region of the protein (Horikami et al., 1994; Chandrika et al., 1995; Holmes and Moyer, 2002; Malur et al., 2002a; Cevik et al., 2003; Smallwood and Moyer, 2004; Dochow et al., 2012). Although this finding spotlights that both the L–P and L–L interaction domains are located in N-terminal regions of the L protein, homo-oligomerization of MeV and SeV L is reportedly independent of P protein binding (Holmes and Moyer, 2002; Cevik et al., 2003; Dochow et al., 2012). The available information is limited, but the specificity for L–P binding apparently involves multiple non-consecutive amino acids that are distinct from those implemented in L–L interactions (Cevik et al., 2004).

## Development of Antiviral Therapeutics

The dynamic interplay between the different viral protein components of the RdRp and the diverse enzymatic activities catalyzed by the L protein constitute an array of drug target candidates suitable for effective inhibition of virus replication. An inherent challenge of all pathogen-directed drug discovery campaigns is a narrow indication spectrum of the therapeutic candidate, limiting inhibitory activity to a specific member or, at best, a single genus within the paramyxovirus family. It may be possible to overcome this restriction by targeting a host cell-derived cofactor of the complex that is likewise indispensible for RdRp activity. For instance, the human translation elongation factor eEF1A is known to be required for VSV RdRp transcriptase activity (Das et al., 1998; Qanungo et al., 2004) and was recently shown to be critically involved also in RSV replication (Wei et al., 2014). A general requirement of eEF1A and/or additional host factors for paramyxovirus RdRp activity is possible, but direct therapeutic targeting of, for instance, eEF1A will likely be prohibited by its central role in host protein synthesis. While it may be hypothetically possible to reduce undesirable cytotoxicity through a campaign specifically designed to block a host cofactor-RdRp protein–protein interaction (PPI), we consider the development of pathogen-directed RdRp blockers more fruitful. Especially “open” high-throughput screening campaigns in search of RdRp inhibitors should yield pathogen-directed hits with higher propensity than compounds interfering with a cofactor-RdRp PPI.

In particular the L protein represents a rich target for drug discovery campaigns, due to its multidomain organization and the concentration of several essential enzymatic activities in a single protein. The L CR-V domain containing a guanylyltransferase domain responsible for 5′-cap formation (Li et al., 2008) is a case in point, since inhibiting the viral capping machinery using guanosine nucleotide analogs constitutes a proven antiviral approach (Lampio et al., 1999; Issur et al., 2011). Likewise, it may be possible to exploit the postulated *S*-adenosyl-L-methionine transferase domain responsible for 5′-cap methylation (Bujnicki and Rychlewski, 2002; Ferron et al., 2002; Ogino et al., 2005; Murphy and Grdzelishvili, 2009) in L CR-VI. *S*-adenosyl-L-homocysteine derivatives have been shown to selectively inhibit methyltransferase activity of dengue virus of the flavivirus family, setting an example for the therapeutic potential of antivirals targeting methyltransferase functions (Lim et al., 2011). The precedence established by the development of inhibitors of, for instance, HIV reverse transcriptase and Hepatitis C virus polymerase underscores the value of high-resolution structural information for the identification and optimization of hit structures, the molecular understanding of the mechanism of inhibitory activity, and, potentially, the proactive design of analogs with increased resilience against viral escape from inhibition (Nijhuis et al., 2009; Adams et al., 2010; Das et al., 2011; Halfon and Locarnini, 2011; Mayhoub, 2012; Lloyd et al., 2014). However, the paramyxovirus drug development field is hampered by the current lack of high-resolution structural information for any mononegavirales L protein. Overcoming this limitation will be a major milestone toward the development of next generation therapeutics.

An envisioned drug application including post-exposure prophylactic use affects the drug profile requested of a desirable anti-paramyxovirus therapeutic; a successful candidate must be safe and efficacious, amenable to cost-effective manufacture, ideally be shelf-stable at ambient temperature, and must be orally bioavailable. Of small-molecule chemical compounds, large molecule biologics, and peptidic biopharmaceutical as candidate drug classes, small-molecules are most suitable to fulfill these divergent demands (Ganellin et al., 2013). Two main classes of polymerase-targeted drugs are currently in clinical use for, among others, antiretroviral therapy, human cytomegalovirus therapy, and HCV therapy, competitive nucleotide/nucleoside substrate analogs and non-nucleoside allosteric inhibitors (Sun et al., 2007, 2008; Andrei et al., 2008; Brown, 2009; Krumm et al., 2011; Mercorelli et al., 2011). **Table 1** provides an overview of some experimental drug candidates targeted against different paramyxovirus RdRps that represent these main classes and are currently under clinical consideration or were found efficacious in animal models of paramyxovirus disease. As discussed below, we consider it the most promising approach to combine, if available, a substrate analog with an allosteric inhibitor to maximize the prospect of capitalizing on drug combination synergies and in particular reduce the frequency of viral escape from inhibition and/or lower the fitness of escape variants with multiple resistance mutations.

**Table 1:** Examples of substrate analog and allosteric paramyxovirus RNA-dependent RNA polymerase (RdRp) inhibitors that showed efficacy in animal models and/or were advanced to clinical trials.

Structure	Name	Indication	Clinical progression
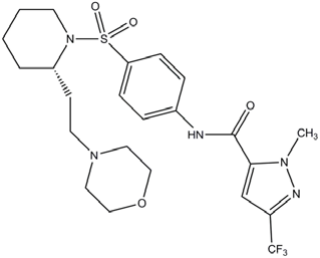	ERDRP-0519	CDV, measles virus (MeV)	Orally efficacious in the ferret-CDV model of morbillivirus disease.

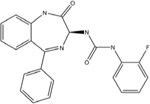	RSV604	Respiratory syncytial virus (RSV)	Phase I–III completed.

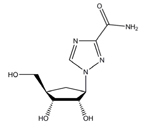	Ribavirin	MeV, mumps virus (MuV), HeV, NIV, RSV hepatitis C, HIV-1, hPIV2, hPIV3, HSV-1, HSV-2, influenza.	FDA approved.

Structure unavailable	ASL-008176	RSV	Reduced viral load in phase II clinical trials in adults.

## Nucleoside and Nucleotide Analogs

Nucleoside analogs contain non-canonical bases that act as chain terminators after intracellular phosphorylation to the corresponding nucleotide and incorporation into the nascent chain (De Clercq and Neyts, 2009; Soriano et al., 2013) While nucleoside analogs have shown extreme clinical success, ribavirin is currently the only substrate analog licensed against a paramyxovirus disease, the treatment of RSV infection. The compound is a purine-analog capable of base-pairing with equal efficiency with either cytosine or uracil (Wright et al., 2005). Rather than acting as a chain terminator, the resulting hypermutation of the newly synthesized strand is considered to block virus replication through error catastrophe (Crotty and Andino, 2002). However, ribavirin efficacy against RSV is limited and severe adverse effects, in particular an increased risk of anemia and mitochondrial toxicity (Canonico, 1985; Gilbert and Knight, 1986; Huggins et al., 1991), undermine its clinical use for anti-RSV therapy. In contrast, ALS-008176, a recently presented novel nucleoside analog, is currently in phase II clinical trials for use against RSV infection (Devincenzo et al., 2014). In this trial, the compound ALS-008176 emerged as well tolerated and was capable of significantly reducing viral load in treated adults compared to the placebo control group, when treatment was initiated at the onset of infection. These data are highly encouraging, since they provide proof-of-concept for the clinical benefit of effective RSV inhibitors. In addition, Favipiravir (T-705), a nucleotide analog investigated for the treatment of several virus infections including influenza A, Ebola virus, and foot-and-mouth-disease virus (Furuta et al., 2002), showed activity against RSV in cell culture, albeit at prohibitively high concentrations for clinical use (Furuta et al., 2002, 2013). A novel nucleoside analog was recently reported as a screening hit emerging from a high-throughput anti-RSV campaign (Laganas et al., 2014). Remarkably, resistance mutations were characterized and mapped to the RSV P protein rather than the L polymerase, suggesting a novel mechanism of antiviral activity that is distinct from chain termination and error catastrophe.

## Non-Nucleoside RdRp Inhibitors

Non-nucleoside allosteric inhibitors non-competitively block RdRp activity through docking into allosteric sites that are frequently located outside of the actual substrate binding site. Binding of an allosteric ligand can either indirectly alter the active site structurally through a long-range effect, rendering the enzyme catalytically inactive, or they may disrupt the formation of protein complexes required for correct enzymatic function. Examples of clinically approved allosteric polymerase inhibitors that served as primary medication in first-line highly active antiretroviral therapy are the first generation non-nucleoside RT inhibitors Nevirapine and Efavirenz (Basavapathruni and Anderson, 2007; de Bethune, 2010). However, the genetic barrier to resistance against these compounds is low and these drugs are unsuitable for monotherapy (Usach et al., 2013). Second-generation non-nucleoside RT inhibitors such as Etravirine and Rilpivirine show improved resistance profiles, allowing use of Etravirine in treatment-experienced patients containing multidrug-resistant HIV (de Bethune, 2010).

Analogous to the experience with non-nucleoside RT inhibitors, the paramyxovirus L protein should present an equally viable target for effective non-nucleoside therapeutics, in particular when used in combination with a nucleoside analog to prevent the induction of genetic drift in the endemic virus populations leading to the development of preexisting resistance.

We have recently developed and mechanistically characterized an allosteric morbillivirus RdRp inhibitor class that targets the L protein based on the experimental induction of escape mutants (**Figure 6**; White et al., 2007; Sun et al., 2008; Yoon et al., 2008, 2009; Krumm et al., 2011; Ndungu et al., 2012; Moore et al., 2013a,b). Specifically, resistance mutations clustered in L protein conserved domains of II, III, and IV (Yoon et al., 2009). Further development of this class yielded the clinical candidate ERDRP-0519, a well-tolerated orally efficacious pan-morbillivirus RdRp inhibitor that rendered normally lethal CDV disease in the ferret model clinically asymptomatic when administered in a post-exposure prophylactic regimen commencing at the onset of viremia (Krumm et al., 2014). Highly encouraging, all post-exposure-treated animals not only survived primary infection but mounted a robust immune response and were completely protected against a subsequent lethal CDV challenge infection (Krumm et al., 2014).

Currently at an early stage of development, several small molecule RSV inhibitors were shown to specifically block RdRp activity in cell culture and show high potential for lead development (Liuzzi et al., 2005; Laganas et al., 2014; Matharu et al., 2014; Tiong-Yip et al., 2014)

In addition to targeting the L protein directly, the paramyxovirus N protein also represents a potential target for viral therapeutics, as evidenced by the recently described RSV inhibitor RSV604 (Chapman et al., 2007). Resistance mutations to RSV604 were hypothesized to include residues involved in the interaction of N with the P–L complex (Chapman et al., 2007). Furthermore, locating resistance hot-spots in RSV N crystal structures revealed important candidate interaction sites, including the RNA binding cavity, the site of N-arm attachment, and the NTD region, which could all also be specifically targeted for the development of therapeutic treatments (Chapman et al., 2007; Tawar et al., 2009; El Omari et al., 2011). Clinical trials have shown that RSV604 was safe and well tolerated by healthy volunteers (Chapman et al., 2007; Marty, 2007; Chapman and Cockerill, 2011; Challa et al., 2014). The compound shows potent antiviral efficacy, using a unique mechanism of action, and is likewise orally bioavailable.

The resistance profile of RSV604 suggests that the compound could possibly interfere with critical PPIs required for RdRp activity. Considering the multitude of dynamic protein–protein contacts required for viral RNA synthesis, specifically targeting protein interfaces such as those between N and P, P and L, P and P, or L and L to block paramyxovirus RdRp represents a currently underexplored opportunity for therapeutic intervention that may hold high future promise.

Short-chain peptides have been explored as candidate inhibitors for a diverse panel of PPIs (DeLano et al., 2000; De Luca et al., 2011; Gavenonis et al., 2014), although poor intracellular availability and rapid proteolysis frequently limit therapeutic use. Small-molecules are more suitable to address these limitations, but until two decades ago, PPIs were essentially considered undruggable by synthetic molecules due to the large (typically 1,000–2000 A^2^) size and flat geometry of the typical PPI interface (Hwang et al., 2010). Subsequently, however, natural small molecule products such as rapamycin and cyclosporine spotlighted that only a subset of residues in small hot-spot areas confers most of the binding energy, making PPIs are amenable to small-molecule docking and interference (Arkin and Wells, 2004; Arkin et al., 2014). In recent years, over 40 PPIs were successfully subjected to small molecule targeting (Higueruelo et al., 2009; Basse et al., 2013; Labbe et al., 2013) and several candidate inhibitors were advanced to clinical testing (Arkin et al., 2014). The precedence set by these advanced PPI blockers demonstrates that PPIs most suitable for therapeutic intervention concentrate hot-spot residues in defined areas of less than 900 A^2^ and binding partners contain short primary sequences (Smith and Gestwicki, 2012; Basse et al., 2013). As our structural insight into the organization of the paramyxovirus RdRp complex and the geometry of the dynamic PPIs advances, well designed screening campaigns should commence with the structure-guided *in silico* evaluation of druggable candidate interfaces, followed by targeted *in silico* and/or high-throughput screens focused on identified suitable PPIs.

## Conclusion

The high contagiousness of paramyxoviruses, the lack of vaccine protection against several clinically highly significant members of the family, and the deliberate decline of vaccination against other family members due to religious believes or concerns about vaccine safety create an urgent need for the development of efficacious paramyxovirus therapeutics. We believe that small-molecule antivirals are best suited to meet the stringent drug profile requested of a successful anti-paramyxovirus drug. The viral polymerase complex in particular presents a rich target for therapeutic interference through competitive substrate analogs and allosteric non-nucleoside inhibitors. The recent advance in the development of PPI inhibitors should furthermore open up the diverse RdRp protein interfaces to therapeutic interference, when more structural insight into the organization of the polymerase complex and its interaction with the NC template becomes available. Considering the challenges associated with rapidly emerging or preexisting viral resistance that we experience in influenza virus monotherapies, drug combination strategies should be explored and, if possible, implemented from the onset of anti-paramyxovirus therapy to reduce the frequency of inducing genetic drift in the endemic virus populations.

## Conflict of Interest Statement

Richard K. Plemper is an inventor on PCT application No. PCT/US05/04565 paramyxovirus family inhibitors and methods of use.
